# Supplementary material for: Combining Medicinal Plant In Vitro Culture with Machine Learning Technologies for Maximizing the Production of Phenolic Compounds
Source: Antioxidants (Basel). 2020 Mar 4;9(3):210. doi: 10.3390/antiox9030210 (PMC7139750; doi:10.3390/antiox9030210)
Supplement: Supplementary file 1 [file antioxidants-09-00210-s001.pdf]

antioxidants

MDPI

Table S1. Salt composition of culture media used in this work.

	Salts	MS (mg L^{-1})	1/2MS (mg L^{-1})
Macronutrients	KNO_3	1900	950
	NH_4NO_3	1650	825
	$CaCl_2 \cdot 2H_2O$	440	220
	$MgSO_4 \cdot 7H_2O$	370	185
	KH_2PO_4	170	85
Micronutrients	$MnSO_4 \cdot 4H_2O$	22.3	
	$ZnSO_4 \cdot 7H_2O$	8.6	
	H_3BO_3	6.2	
	KI	0.83	
	$CuSO_4 \cdot 5H_2O$	0.025	
	$Na_2MoO_4 \cdot 2H_2O$	0.25	
	$CoCl_2 \cdot 6H_2O$	0.025	
	Na_2EDTA	37.25	
	$FeSO_4 \cdot 7H_2O$	27.85	

Table S2. Dataset used for the construction of ANN model. Inputs: "Gen" refers to genotype, "A" stands for "Aerial parts" and "R" stands for "Roots". Outputs: TPC, FC and RSA are expressed as the mean ± standard deviation of three independent extracts.

Inputs											Outputs		
Genot.	Organ	Solvent	NO_3^-	NH_4^+	K^+	Cl^-	Ca^{2+}	Mg^{2+}	HPO_4^{2-}	SO_4^{2-}	TPC	FC	RSA
BH	A	M40	39.4	20.6	20.0	5.99	2.99	1.50	1.25	1.76	11.0 ± 0.2	0.8 ± 0.02	4.1 ± 0.2
BH	A	M60	39.4	20.6	20.0	5.99	2.99	1.50	1.25	1.76	17.1 ± 0.6	1.2 ± 0.1	2.8 ± 0.2
BH	A	M80	39.4	20.6	20.0	5.99	2.99	1.50	1.25	1.76	22.5 ± 0.6	5.6 ± 0.3	2.0 ± 0.1
BH	A	M100	39.4	20.6	20.0	5.99	2.99	1.50	1.25	1.76	15.1 ± 0.2	9.2 ± 0.2	3.8 ± 0.1
BH	A	M40	19.7	10.3	10.0	2.99	1.50	0.75	0.62	1.01	22.0 ± 0.8	1.0 ± 0.1	2.6 ± 0.04
BH	A	M60	19.7	10.3	10.0	2.99	1.50	0.75	0.62	1.01	37.6 ± 1.4	1.8 ± 0.1	1.2 ± 0.01
BH	A	M80	19.7	10.3	10.0	2.99	1.50	0.75	0.62	1.01	50.0 ± 1.7	5.9 ± 0.1	0.8 ± 0.07
BH	A	M100	19.7	10.3	10.0	2.99	1.50	0.75	0.62	1.01	30.8 ± 0.8	9.9 ± 0.2	1.5 ± 0.1
BH	R	M40	39.4	20.6	20.0	5.99	2.99	1.50	1.25	1.76	7.1 ± 0.3	0.4 ± 0.03	8.1 ± 0.1
BH	R	M60	39.4	20.6	20.0	5.99	2.99	1.50	1.25	1.76	14.0 ± 0.2	0.7 ± 0.02	4.2 ± 0.1

BH	R	M80	39.4	20.6	20.0	5.99	2.99	1.50	1.25	1.76	8.9 ± 0.3	1.5 ± 0.1	5.0 ± 0.1
BH	R	M100	39.4	20.6	20.0	5.99	2.99	1.50	1.25	1.76	9.3 ± 0.2	1.8 ± 0.1	6.0 ± 0.03
BH	R	M40	19.7	10.3	10.0	2.99	1.50	0.75	0.62	1.01	14.1 ± 0.5	0.9 ± 0.02	3.5 ± 0.1
BH	R	M60	19.7	10.3	10.0	2.99	1.50	0.75	0.62	1.01	14.9 ± 0.5	0.9 ± 0.04	3.8 ± 0.1
BH	R	M80	19.7	10.3	10.0	2.99	1.50	0.75	0.62	1.01	13.4 ± 0.3	1.6 ± 0.1	3.7 ± 0.04
BH	R	M100	19.7	10.3	10.0	2.99	1.50	0.75	0.62	1.01	11.8 ± 0.3	1.7 ± 0.04	5.2 ± 0.1
BD	A	M40	39.4	20.6	20.0	5.99	2.99	1.50	1.25	1.76	8.3 ± 0.2	1.8 ± 0.1	5.4 ± 0.2
BD	A	M60	39.4	20.6	20.0	5.99	2.99	1.50	1.25	1.76	20.8 ± 0.5	3.9 ± 0.1	2.7 ± 0.03
BD	A	M80	39.4	20.6	20.0	5.99	2.99	1.50	1.25	1.76	19.9 ± 0.5	10.2 ± 0.4	2.7 ± 0.1
BD	A	M100	39.4	20.6	20.0	5.99	2.99	1.50	1.25	1.76	18.3 ± 0.5	16.0 ± 0.3	3.2 ± 0.03
BD	A	M40	19.7	10.3	10.0	2.99	1.50	0.75	0.62	1.01	14.4 ± 0.4	2.7 ± 0.1	3.7 ± 0.1
BD	A	M60	19.7	10.3	10.0	2.99	1.50	0.75	0.62	1.01	23.6 ± 0.3	3.7 ± 0.1	2.0 ± 0.1
BD	A	M80	19.7	10.3	10.0	2.99	1.50	0.75	0.62	1.01	31.1 ± 0.7	7.3 ± 0.2	1.6 ± 0.05
BD	A	M100	19.7	10.3	10.0	2.99	1.50	0.75	0.62	1.01	25.3 ± 0.2	15.8 ± 0.4	2.3 ± 0.1
BD	R	M40	39.4	20.6	20.0	5.99	2.99	1.50	1.25	1.76	10.2 ± 0.6	0.4 ± 0.01	5.0 ± 0.2
BD	R	M60	39.4	20.6	20.0	5.99	2.99	1.50	1.25	1.76	19.5 ± 0.5	1.0 ± 0.03	2.6 ± 0.1
BD	R	M80	39.4	20.6	20.0	5.99	2.99	1.50	1.25	1.76	16.0 ± 0.4	1.4 ± 0.04	3.3 ± 0.1
BD	R	M100	39.4	20.6	20.0	5.99	2.99	1.50	1.25	1.76	13.5 ± 0.4	2.8 ± 0.1	4.6 ± 0.1
BD	R	M40	19.7	10.3	10.0	2.99	1.50	0.75	0.62	1.01	10.7 ± 0.4	0.6 ± 0.1	4.4 ± 0.2
BD	R	M60	19.7	10.3	10.0	2.99	1.50	0.75	0.62	1.01	19.6 ± 0.7	1.1 ± 0.04	2.4 ± 0.1
BD	R	M80	19.7	10.3	10.0	2.99	1.50	0.75	0.62	1.01	16.5 ± 0.2	2.9 ± 0.1	3.0 ± 0.1
BD	R	M100	19.7	10.3	10.0	2.99	1.50	0.75	0.62	1.01	13.6 ± 0.5	2.6 ± 0.1	4.0 ± 0.1
BT	A	M40	39.4	20.6	20.0	5.99	2.99	1.50	1.25	1.76	7.7 ± 0.3	0.4 ± 0.02	5.6 ± 0.1
BT	A	M60	39.4	20.6	20.0	5.99	2.99	1.50	1.25	1.76	17.7 ± 1.1	1.5 ± 0.02	2.6 ± 0.1
BT	A	M80	39.4	20.6	20.0	5.99	2.99	1.50	1.25	1.76	12.5 ± 0.7	5.4 ± 0.3	4.0 ± 0.2
BT	A	M100	39.4	20.6	20.0	5.99	2.99	1.50	1.25	1.76	10.5 ± 0.2	9.3 ± 0.4	4.5 ± 0.1
BT	A	M40	19.7	10.3	10.0	2.99	1.50	0.75	0.62	1.01	17.1 ± 0.3	0.5 ± 0.002	3.1 ± 0.1
BT	A	M60	19.7	10.3	10.0	2.99	1.50	0.75	0.62	1.01	25.6 ± 0.8	1.4 ± 0.03	1.7 ± 0.01
BT	A	M80	19.7	10.3	10.0	2.99	1.50	0.75	0.62	1.01	15.7 ± 1.0	7.4 ± 0.1	3.3 ± 0.1
BT	A	M100	19.7	10.3	10.0	2.99	1.50	0.75	0.62	1.01	14.4 ± 0.3	9.7 ± 0.2	4.3 ± 0.2
BT	R	M40	39.4	20.6	20.0	5.99	2.99	1.50	1.25	1.76	9.4 ± 0.3	0.5 ± 0.04	5.4 ± 0.1
BT	R	M60	39.4	20.6	20.0	5.99	2.99	1.50	1.25	1.76	11.6 ± 0.2	0.7 ± 0.01	5.0 ± 0.1
BT	R	M80	39.4	20.6	20.0	5.99	2.99	1.50	1.25	1.76	9.2 ± 0.3	1.5 ± 0.02	5.6 ± 0.1
BT	R	M100	39.4	20.6	20.0	5.99	2.99	1.50	1.25	1.76	7.3 ± 0.2	1.6 ± 0.1	9.3 ± 0.4
BT	R	M40	19.7	10.3	10.0	2.99	1.50	0.75	0.62	1.01	13.2 ± 0.1	0.9 ± 0.1	3.8 ± 0.1
BT	R	M60	19.7	10.3	10.0	2.99	1.50	0.75	0.62	1.01	15.0 ± 0.3	1.0 ± 0.01	3.3 ± 0.1
BT	R	M80	19.7	10.3	10.0	2.99	1.50	0.75	0.62	1.01	13.3 ± 0.1	1.6 ± 0.05	3.5 ± 0.1
BT	R	M100	19.7	10.3	10.0	2.99	1.50	0.75	0.62	1.01	10.7 ± 0.5	2.1 ± 0.01	5.6 ± 0.3

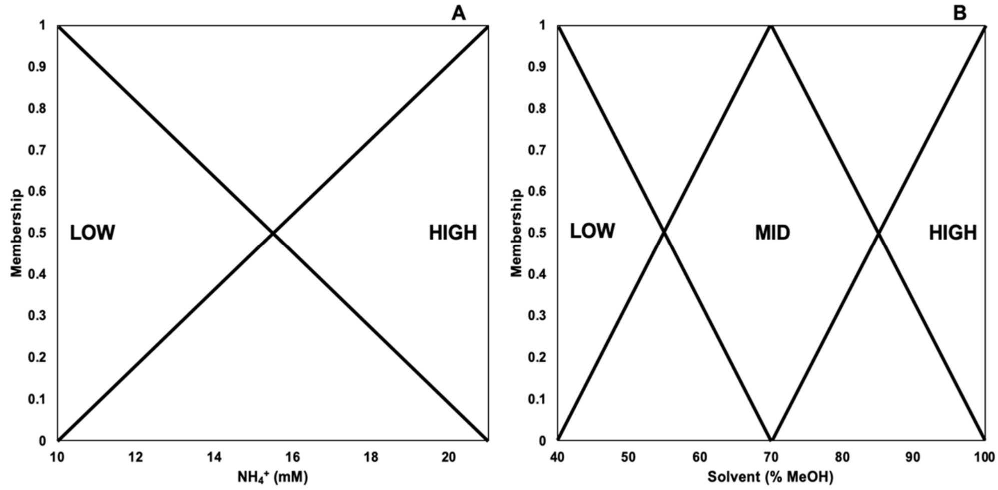

Figure S1. Graphical interpretation of input levels ranged by neurofuzzy logic on TPC modelling: (**A**) NH_4^+ concentration, expressed in mM; (**B**) Methanol proportion in the solvent, expressed as % MeOH.

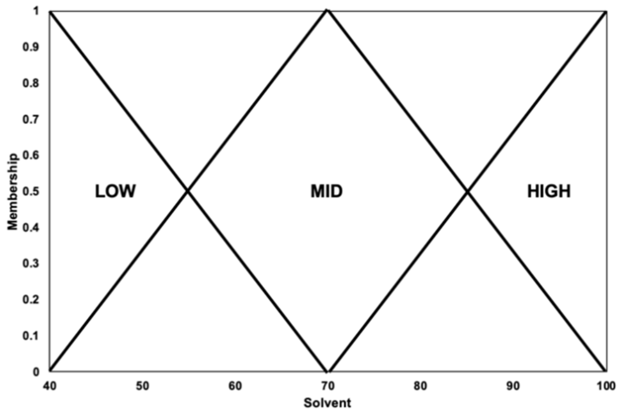

Figure S2. Graphical interpretation of solvent levels ranged by neurofuzzy logic on FC modelling. Results were expressed as % MeOH.

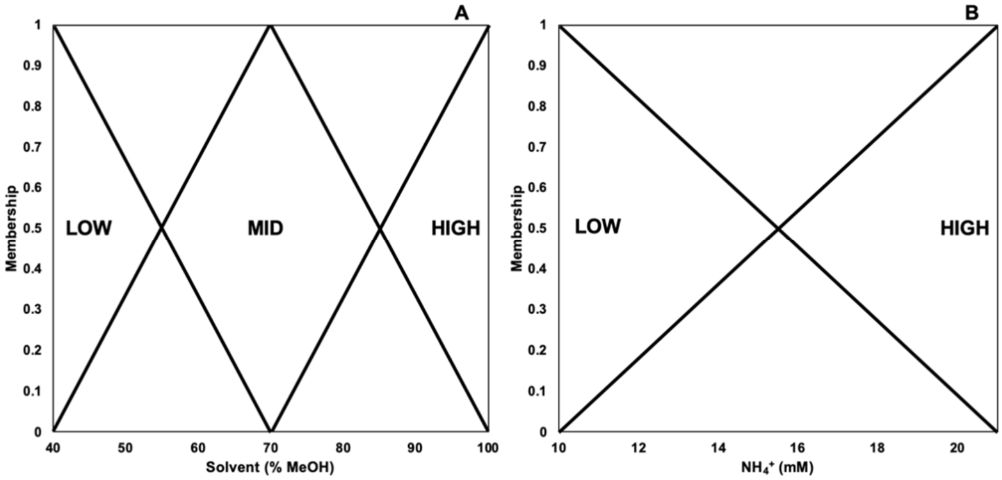

Figure S3. Graphical interpretation of input levels ranged by neurofuzzy logic on RSA modelling: (**A**) Methanol proportion in the solvent, expressed as % MeOH; (**B**) NH_4^+ concentration, expressed in mM.
